# From the Lab to the Field: Potential Applications of Dry EEG Systems to Understand the Brain-Behavior Relationship in Sports

**DOI:** 10.3389/fnins.2019.00893

**Published:** 2019-08-27

**Authors:** Chun-Hao Wang, David Moreau, Shih-Chun Kao

**Affiliations:** ^1^Institute of Physical Education, Health & Leisure Studies, National Cheng Kung University, Tainan, Taiwan; ^2^School of Psychology and Centre for Brain Research, University of Auckland, Auckland, New Zealand; ^3^Department of Health and Kinesiology, Purdue University, West Lafayette, IN, United States

**Keywords:** dry electrodes, neural dynamics, sports expertise, sports neuroscience, mobile EEG system

## EEG as a Window to the Brain Dynamics of Expert Behavior in Sports

Our knowledge of perceptual-cognitive expertise in sports has grown considerably over recent decades (Williams and Ford, 2008). Superior performance in a variety of lab-based cognitive measurements such as cognitive control (Wang et al., 2013; Bianco et al., 2017b; Wylie et al., 2018), spatial ability (Moreau, 2013b; Wang et al., 2015), working memory (Moreau, 2013a), and selective attention (Hung et al., 2004; Alves et al., 2013) have been consistently associated with sports expertise. Further, empirical studies have demonstrated the potential predictive power of cognitive skills (e.g., executive function) to real-world athletic performance (Vestberg et al., 2012, 2017; Cona et al., 2015). Taken together, these behavioral findings highlight the relationship of context-general cognition with sports.

The rapid development of neuroimaging techniques has greatly increased our understanding of the neural correlates underlying the link between cognitive skills and sport expertise in lab-based research (Yarrow et al., 2009; Cheron et al., 2016). Electroencephalography (EEG), a non-invasive and real-time measure of the brain's electrical potentials (Biasiucci et al., 2019), is fairly suitable for investigating cortical activity in both laboratory and field conditions as compared to other neuroimaging techniques such as functional magnetic resonance imaging (fMRI), probably due to its compact size and low cost (Mehta and Parasuraman, 2013). Specifically, the high temporal resolution of EEG offers a particularly precise means to examine the rapid time course of cognitive processes inherent to sporting behavior (Nakata et al., 2010; Park et al., 2015). A number of EEG studies have demonstrated that sports experts display more effective neural modulations in cognitive paradigms irrelevant to any sport-related contexts (Hung et al., 2004; Di Russo et al., 2006; Nakata et al., 2010; Wang et al., 2015; Bianco et al., 2017a), especially for those practicing a sport requiring both physical and cognitive demands (Yamashiro et al., 2015; Wang et al., 2017). Using concurrent EEG recording with execution of cognitive or motor skills relevant to sport expertise, previous studies revealed more efficient task-related cortical activation as a function of inter-individual expertise level or intra-individual performance level (Di Russo et al., 2005; Del Percio et al., 2008, 2009; Babiloni et al., 2010; Wang and Tu, 2017). As such, EEG has been proposed as a neural measure to characterize sporting performance states in elite athletes (e.g., Bertollo et al., 2016; Di Fronso et al., 2016). Further, research exploring EEG signatures of expert performance in sports has led to empirical studies investigating the modifiability of neuromarkers linked to optimal sporting performance through neurofeedback training (NFT) (Mirifar et al., 2017; Xiang et al., 2018). This line of research has not only explored the potential mediating role of EEG correlates in the relationship between sports training and performance but also promoted the development of multimodal training combining sports and neurosciences.

Despite the growing number of EEG research on brain functioning of athletes, the limited portability and long preparation time of conventional EEG systems have been the frequently cited limitations that made EEG assessment unpractical beyond laboratory settings. Recent progress in EEG technologies has offered potential solutions by improving the flexibility in mobile EEG applications (i.e., portable, wireless, and disposable headset) (Makeig et al., 2009; Mehta and Parasuraman, 2013; Oliveira et al., 2016; Bateson et al., 2017; Lau-Zhu et al., 2019). While the ecologically friendly EEG systems with wet electrodes allow the collection of good quality signals during natural motion, they present a number of drawbacks that may impede the real-world application in a wide range of environments (Zander et al., 2011). For example, gel application, skin preparation, and post-record cleaning are time-consuming and uncomfortable for participants. Prolonged and repeated data collection may also induce allergic reactions or infections. Moreover, frequent gel replacement due to gel drying is necessary to ensure the quality of EEG signals, making long-term monitoring more challenging.

To overcome the limitation inherent to wet EEG assessments, dry EEG systems have been developed over the past few years (Chen et al., 2014; Lopez-Gordo et al., 2014). In this brief review, we present the potential utility of dry EEG systems to facilitate the progression from lab-based research to field applications from the perspective of sport and exercise sciences. For a thorough review of the fundamental differences between wet and dry EEG systems, readers are referred to prior literature (Chi et al., 2010; Xu et al., 2017).

## Possible Utility of dry EEG Systems in Moving Sports Neuroscience TOWARD Real-World Measurements

EEG oscillations in theta, alpha and beta frequency ranges have been considered as important indices of cognitive and motor processing in athletes (Babiloni et al., 2008; Del Percio et al., 2009; Nakata et al., 2010; Kao et al., 2013). With regard to existing evidence observed across studies with varying levels of ecological validity, it is possible to bridge the gap between lab-based findings and field-based practice. As an example, expert athletes practicing sports involving complex motor skills have been shown to exhibit greater modulation of theta oscillations (Wang et al., 2015, 2017) that is functionally coupled with attentional control processes during lab-based cognitive tasks (Nigbur et al., 2012; Cavanagh and Frank, 2014). Interestingly, oscillatory theta activity has also been associated with expert-novice differences in motor skills (Baumeister et al., 2008) and behavioral performance during motor tasks mimicking sports specific skills (Chuang et al., 2013; Kao et al., 2013). Further, research suggested that frontal theta oscillations are modifiable through NFT (Enriquez-Geppert et al., 2014), which was found to improve attentional (Wang and Hsieh, 2013) and skill performance (Kao et al., 2014). However, the investigations of such neuroelectric profiles in artificial environments would only further our understanding of the brain functioning associated with sports expertise in these specific circumstances, but not necessarily generalize to natural sports environments. Moreover, although quite a few studies have examined self-paced, closed-skilled sports in more ecologically valid conditions (Deeny et al., 2009; Nakata et al., 2010; Babiloni et al., 2011; Cheng et al., 2015), these are limited to conditions requiring very little movement such as the pre-performance period (Hatfield et al., 2004). The paucity of studies using conventional recording systems that investigate complex motor skills in novel environments may be due in part to the lack of flexibility and the vulnerability to various non-neural artifacts (Thompson et al., 2008; Mullen et al., 2015; Park et al., 2015).

While the traditional Ag/AgCl electrodes with wet conductive gel have the capacity to maintain low skin-electrode impedance during movement, remaining issues of using wet electrodes such as time-consuming skin preparation and uncomfortable removal are also well-known (Liao et al., 2011; Chen et al., 2014). Furthermore, issues with long-term monitoring such as signal degradation due to gel drying and skin irritation are often observed. Recently, dry electrodes have been developed in an attempt to overcome the drawbacks of wet systems (Chen et al., 2014; Xu et al., 2017). For example, thanks to the gel-free operation, dry electrodes enable a faster setup time (Lopez-Gordo et al., 2014; Xu et al., 2017). This may be particularly beneficial when investigating acute neurocognitive effects following sport or exercise training given that the exercise-induced transient changes in cognitive function are time-sensitive (Chang et al., 2012; Moreau and Chou, 2019). Compared to the fitting and removal of wet electrodes, which require specific expertise (Chen et al., 2014), the easy-to-handle dry electrodes can be quite helpful in fostering the practical applications of EEG in sports while minimizing variances related to measurement errors (e.g., cross-talk between electrodes due to excessive amounts of gel application). The aforementioned advantages of dry electrodes would improve the feasibility of continuously monitoring temporal dynamics of brain functioning during a training session, cognitive readiness in response to pre-competition warm-up activities, or cognitive recovery following post-exercise cool-down activities.

Recent evidence, albeit limited, has shown reliable cognitive EEG signals such as the P300 component of event-related potentials (EPRs) using dry electrode sensors (Zander et al., 2011; Mathewson et al., 2017). P300 is commonly thought to reflect attention allocated to a given task (Polich, 2007; Wang et al., 2016; Kao et al., 2019) and has been associated with sports expertise (Nakata et al., 2010; Zhang et al., 2015; Wang and Tu, 2017). Thus, the investigation of P300 using dry systems may provide information complementary to that of conventional EEG systems. In the study by Zander et al. (2011), the authors found that the peak amplitude of P300 elicited during an oddball paradigm did not differ significantly between wet and dry systems. Moreover, Debener et al. (2012) observed reliable P300 during a auditory oddball paradigm while participants walked around campus, as well as in seated, indoor conditions. The results revealed that single-trial P300 could be successfully classified (classification accuracy: 69%, ranging from 54 to 88%), with further correlation analysis showing a strong association of P300 amplitude between indoor and outdoor conditions. A follow-up study by De Vos et al. (2014) replicated the P300 results by revealing classification accuracy greater than chance-level in both outdoor seated and walking recording, demonstrating that dry electrodes can tolerate reasonable gross movements in realistic daily life scenarios. However, it is worth noting that Oliveira et al. (2016) found predominantly inferior EEG quality including higher rejection rate, pre-stimulus noise, ERP amplitude variance, and lower signal-to-noise ratio (SNR) in comparison to wet systems in both seated and walking conditions. Despite the fact that this discrepancy might be due to the differences in methods used across different studies (e.g., channel selection for analysis), Oliveira et al. (2016) suggested that dry systems may need substantial improvement to meet the data quality from wet systems. Nonetheless, the available evidence suggests that dry electrode systems may be a promising approach for the transition from the labs to ecological environments.

## Future Prospects and Challenges

Given the promising results of dry EEG systems discussed above, an important development will be the extension of existing EEG evidence in the context of sports expertise to real-world sports environments, such as using EEG to complement evaluations of optimal performance, expertise level, skill development, the effectiveness of training regimens, or even talent identification in sports. In terms of field-based applications, EEG activity has been found to change as a function of exercise intensity (Kamijo et al., 2004) or exercise modality (Kao et al., 2017, 2018), and may also reflect exercise-induced fatigue (Vargas and Marino, 2014). Thus, it would be of interest to explore whether a sport-specific warm-up exercise could more effectively activate the brain networks underlying complex motor skill acquisition or motor execution relative to a general warm-up exercise, which would help to refine the parameters of a warm-up protocol for a specific sport. Moreover, multi-methods data collections such as the simultaneous recording and integration of EEG and other physiological data would provide complementary information about the effectiveness of a training program, such as monitoring whether the dosage (i.e., intensity, duration, frequency) of training is mentally and physically sufficient. More pointedly, given the utility of mobile cognition approach in understanding cognitive operations in contexts related to the natural interaction within the environment (Ladouce et al., 2017), it would be feasible to take the advantage of user-friendly mobile applications of dry sensors to characterize the neural profiles of expert performance during execution of complex sports skills. Finally, more attention has recently been directed toward the meditating role of cognitive function in sports success (Cona et al., 2015; Vestberg et al., 2017), which thus warrants further steps toward the development of a comprehensive cognitive assessment combined with concurrent mobile dry EEG recording by observing cognitive and brain dynamics, as both may help uncover additional mechanisms behind fluctuation of sports performance efficiency.

Although promising, the utility of dry EEG electrodes in understanding natural behavior in sports is still in its infancy, with a number of potential limitations. First, although dry electrodes have been shown to provide reliable measures of EEG (Debener et al., 2012; De Vos et al., 2014), as evidenced by a valid level of classification accuracy (De Vos et al., 2014), a broad-band increase in EEG power compared to that recorded from wet electrodes was observed within the same experimental condition (Mathewson et al., 2017). Future studies should consider the possible influence of calibration settings or electrode materials (Mathewson et al., 2017), which may be responsible for these discrepancies. Second, previous studies have reported a significant drop in classification accuracy levels for the single-trial analysis during walking outdoor conditions, relative to a seated indoor (Debener et al., 2012) or a seated outdoor recording condition (De Vos et al., 2014). This issue is particularly important given that dry electrodes may be more susceptible to movement artifacts and produce higher levels of noise (e.g., lower S-R ratio) than gel based-systems (Oliveira et al., 2016; Radüntz, 2018), which may in turn result in a decrease in statistical power (Mathewson et al., 2017). Although a five-fold increase in trial numbers may help detect the effects of interest (Mathewson et al., 2017), doing so could offset the ease of application of dry electrodes as previously recommended (e.g., shorter setup time). Furthermore, to ensure good skin contact during data collection using dry electrodes (Lau-Zhu et al., 2019), the combination of rigid electrodes and increased pressure can result in discomfort (Oliveira et al., 2016), which in turn can affect cognitive EEG activities (Lorenz and Bromm, 1997). Soft electrodes such as silver-coated polymer bristles (Grozea et al., 2011), dry foam electrodes (Lin et al., 2010), and polymer-based electrodes (Chen et al., 2014) seem to be promising alternatives for mobile EEG applications while maintaining low impedance (Xu et al., 2017). Further, the non-contact systems which isolate the electrodes and the scalp by capacitive coupling (Chi et al., 2010) may also be useful in mitigating this issue, despite some major drawbacks that should be considered with caution (Lopez-Gordo et al., 2014). Taken together, we believe that the emerging techniques of dry EEG systems will offer additional value on top of evidence from traditional recording systems to further understand the brain-behavior relationship in sports.

## Concluding Remarks

Growing literature has demonstrated the utility of dry EEG electrodes in the research on neuroelectric underpinnings that underlie perceptual-cognitive processes in field-based research. As such, dry EEG systems may have the potential to help better understand and monitor brain dynamics during sports performance, in response to training, and under sport-specific contexts. The majority of existing evidence derived from traditional laboratory research has provided a strong rationale for future studies using dry EEG systems to target certain EEG correlates such as frontal theta oscillations or P300-ERP that have been found to reflect optimal perceptual-cognitive states underlying sports expertise. Despite some limitations, we believe that the rapid development of dry EEG techniques can improve knowledge translation in sports neuroscience research and practice.

## Author Contributions

All authors listed have made a substantial, direct and intellectual contribution to the work, and approved it for publication.

### Conflict of Interest Statement

The authors declare that the research was conducted in the absence of any commercial or financial relationships that could be construed as a potential conflict of interest.
